# Supplementary material for: Feasibility of a conversation-based brief intervention in general practice to reduce post-traumatic symptoms after intensive care treatment—A qualitative analysis of the PICTURE study
Source: PLOS Ment Health. 2026 Jan 28;3(1):e0000467. doi: 10.1371/journal.pmen.0000467 (PMC12851445; doi:10.1371/journal.pmen.0000467)
Supplement: S1 File — (PDF) [file pmen.0000467.s001.pdf]

Acknowledgements

Our thanks go to the German Center for Mental Health (DZPG), the Bavarian Research Practice Network (BayFoNet) and the Research Practice Network Berlin-BrandenburgThuringia (RESPoNsE).

PICTURE Study Group, in alphabetical order:

Adrion, Christine4; Angstwurm, Matthias5; Bergmann, Antje6; Beutel, Antina1; Bielmeier, Gerhard7; Bischhoff, Andrea1; Bogdanski, Ralph8; Brettner, Franz9; Brettschneider, Christian10; Briegel, Josef11; Bürkle, Martin12; Dohmann, Johanna1; Elbert, Thomas3; Falkai, Peter13; Felbinger, Thomas 14; Fisch, Richard15; Förstl, Hans16; Fohr, Benjamin17; Franz, Martin18; Friederich, Patrick15 Friemel, Chris-Maria 1; Gallinat, Jürgen19; Gehrke-Beck, Sabine2; Gensichen, Jochen1; Gerlach, Herwig18; Güldner, Andreas20; Hardt, Hanna21; Heintze, Christoph2; Heinz, Andreas22; Heller, Axel23; von Heymann, Christian18; Hoppmann, Petra24; Huge, Volker25; Irlbeck, Michael11; Jaschinski, Ulrich23; Jarczak, Dominik26; Joos, Stefanie27; Kaiser, Elisabeth3; Kerinn, Melanie26; Klefisch, Frank-Rainer28; Kluge, Stefan24; Koch, Roland27; Koch, Thea20; Kowalski, Michelle2; König, Hans-Helmut10; Kosilek, Robert Philipp1; Lackermeier, Peter12; Laugwitz, Karl-Ludwig24; Le, Tri2 Lemke, Yvonne26; Lies, Achim18; Linde, Klaus29; Lindemann, Daniela1; Lühmann, Dagmar21; Lukaschek, Karoline1; May, Stephanie20; Ney, Ludwig11; Oltrogge, Jan21; Pankow, Wulf18; Papiol, Sergi30; Ragaller, Maximilian20; Rank, Nikolaus7; Reill, Lorenz18; Reips, Ulf-Dietrich3; Richter, Hans-Peter31; Riessen, Reimer32; Ringeis, Grit26; Rüchhardt, Ann1; Sanftenberg, Linda1;Schauer, Maggie3; Schelling, Gustav11; Schelling, Jörg1; Scherag, André33; Scherer, Martin21; Schubert, Tomke2; Schmidt, Konrad2; Schneider, Antonius29; Schneider, Gerhard8; Schneider, Jürgen8; Schnurr, Julia27; Schultz, Susanne1; Schulze, Thomas G30.; Schumacher, Karin1; Singhammer, John37, Spieth, Peter20; Theisen, Kerstin2; Thurm, Franka 33; Vogl, Thomas 35; Voigt, Karen6; Walther, Andreas17; Wassilowsky, Dietmar11; Wäscher, Cornelia2; ; Wehrstedt, Regina1; Weiss, Björn38; Weierstall-Pust, Roland36; Weis, Marion11; Weiss, Georg12; Well, Harald12; Zöllner, Christian26; Zwissler, Bernhard11

Affiliations

1 LMU University Hospital, Institute of General Practice and Family Medicine, Munich, Germany

2 Charité – Universitätsmedizin Berlin, Institute of General Practice and Family Medicine, Berlin, Germany

3 Department of Psychology, University of Konstanz, Konstanz, Germany

4 Institute for Medical Information Processing, Biometry, and Epidemiology (IBE), LMU Munich, Munich, Germany

5 LMU University Hospital, Medical Clinic IV, Munich, Germany

6 Department of General Practice/Clinic of General Medicine – Medical clinic III, University Hospital Carl Gustav Carus, Technische Universität Dresden, Dresden, Germany

7 Dritter Orden Clinic, Munich, Germany

8 Technical University of Munich, School of Medicine, Klinikum rechts der Isar, Department of Anesthesiology and Intensive Care, Munich, Germany

9 Brothers of Mercy Hospital Munich, Clinic of Anesthesiology and Intensive Care, Munich, Germany

10 Department of Health Economics and Health Services Research, University Medical Center Hamburg-Eppendorf, Hamburg, Germany

11 Department of Anaesthesiology, University Hospital, LMU Munich, Munich, Germany

12 Isarklinikum Anästhesie, Munich, Germany

13 Clinic for Psychiatry and Psychotherapy, LMU University Hospital, Munich, Germany

14 Department of Anesthesiology, Critical Care and Pain Medicine, Harlaching Medical Center, The Munich Municipal Hospitals Ltd, Munich, Germany

15 Department of Anesthesiology, Critical Care and Pain Medicine, Bogenhausen Medical Center, The Munich Municipal Hospitals Ltd, Munich, Germany

16 Department of Psychiatry and Psychotherapy, Technical University of Munich, München, Germany

17 Department of Anaesthesiology and operative Intensive Care, Klinikum Stuttgart, Stuttgart, Germany

18 Clinic for Anesthesiology, Operative Intensive Care and Pain Management, Vivantes Klinikum Neukölln, Berlin, Germany

19 Department of Psychiatry and Psychotherapy of the University Medical Center Hamburg-Eppendorf, Hamburg, Germany

20 Clinic of Anesthesiology and Intensive Care Medicine, University Hospital Carl Gustav Carus, Technische Universität Dresden, Dresden, Germany

21 Department of General Practice / Primary Care, University Medical Center Hamburg-Eppendorf, Hamburg, Germany

22 Department of Psychiatry and Psychotherapy Campus Charité Mitte, Berlin, Germany

23 Clinic of Anesthesiology and Intensive Care Medicine, Augsburg University, Augsburg, Germany

24 Department of Internal Medicine, Technical University of Munich, Klinikum rechts der Isar, München, Germany

25 Intensive Care Medicine, Schoen Clinic Bad Aibling Harthausen, Schoen Clinic Group, Munich, Germany

26 Center for Anesthesiology and Intensive Care Medicine, University Medical Center Hamburg-Eppendorf, Hamburg, Germany.

27 Institute for General Practice and Interprofessional Health Care, University Clinic Tübingen, Tübingen, Germany

28 Intensive Care Unit, Paulinenkrankenhaus, Berlin, Germany

29 Institute of General Practice, Technical University of Munich, Klinikum rechts der Isar, Munich, Germany

30 Institute of Psychiatric Phenomics and Genomics, University Hospital, LMU Munich, Munich, Germany

31 Department of Cardiology, Pneumology and Internal Intensive Care Medicine, Schwabing Medical Center, The Munich Municipal Hospitals Ltd, Munich, Germany

32 Department of internal intensive care, University Clinic Tübingen, Germany

33 Institute of Medical Statistics, Computer and Data Sciences, Jena University Hospital, Jena, Germany

34 Chair of lifespan developmental neuroscience, University Hospital Carl Gustav Carus, Technische Universität Dresden, Dresden, Germany

35 Institute of Diagnostic and Interventional Radiology, Universityhospital Frankfurt, Frankfurt am Main, Germany

36 MSH Medical School Hamburg University of Applied Sciences and Medical University, Hamburg, Germany

37 Fresenius University of Applied Sciences, Idstein, Germany

38 Department of Anesthesiology and Intensive Care Medicine, Charité University Medicine, Berlin, Germany
