# Supplementary material for: Feasibility of a conversation-based brief intervention in general practice to reduce post-traumatic symptoms after intensive care treatment—A qualitative analysis of the PICTURE study
Source: PLOS Ment Health. 2026 Jan 28;3(1):e0000467. doi: 10.1371/journal.pmen.0000467 (PMC12851445; doi:10.1371/journal.pmen.0000467)
Supplement: S2 Text — (PDF) [file pmen.0000467.s002.pdf]

Interviewer:

Date:

GP carrying out the intervention:

Patient ID:

Feasibility (duration, problems, acceptance by the patient)	
Preparation through the training (competence, open questions?)	
Effectiveness (Did it help the patient?)	
Usefulness/transferability (applicability to other patients, barriers?)	
Further notes	
