# Supplementary material for: Feasibility of a conversation-based brief intervention in general practice to reduce post-traumatic symptoms after intensive care treatment—A qualitative analysis of the PICTURE study
Source: PLOS Ment Health. 2026 Jan 28;3(1):e0000467. doi: 10.1371/journal.pmen.0000467 (PMC12851445; doi:10.1371/journal.pmen.0000467)
Supplement: S3 Checklist — (PDF) [file pmen.0000467.s003.pdf]

COREQ (COnsolidated criteria for REporting Qualitative research) Checklist

(Tong A, Sainsbury P, Craig J. COREQ checklist for interviews and focus groups. Int J Qual Health Care. 2007;19(6):349-57.)

Topic	Item No.	Guide Questions/Description	Reported on Page No./Notes
**Domain 1: Research team and reflexivity**			
Personal characteristics			
Interviewer/facilitator	1	Which author/s conducted the interview or focus group?	p.7: AB and CF conducted the interviews.
Credentials	2	What were the researcher's credentials? E.g. PhD, MD	p.7: CF is a psychologist and AB was at the time of data collection a master's student in psychology and also an experienced nurse.
Occupation	3	What was their occupation at the time of the study?	See section above.
Gender	4	Was the researcher male or female?	AB is female.
Experience and training	5	What experience or training did the researcher have?	p.8: They had ongoing collegial exchange with the PICTURE team at the Munich Study Center and in a qualitative research seminar at LMU Munich.

Relationship with participants

Relationship established	6	Was a relationship established prior to study commencement?	p.7: No relationship prior to study commencement.
Participant knowledge of the interviewer	7	What did the participants know about the researcher? e.g. personal goals, reasons for doing the research	p.7: The interviewees were informed about the professions of the callers and the aim of the survey during the study.
Interviewer characteristics	8	What characteristics were reported about the interviewer/facilitator? e.g. Bias, assumptions, reasons and interests in the research topic	p.7: The interviewees were informed about the content of the calls when the appointments were made.

Domain 2: Study design

Theoretical framework

Methodological orientation and Theory	9	What methodological orientation was stated to underpin the study? e.g. grounded theory, discourse analysis, ethnography, phenomenology, content analysis	p.8: The first author conducted a structuring qualitative content analysis based on Kuckartz (2018).

Sampling	10	How were participants selected? e.g. purposive, convenience, consecutive, snowball	p.7: The GP participants in the main PICTURE study were not selected but were asked to participate after their patients had been included in the study. Alternative GPs were recruited by the study team for those patients whose GPs had declined to participate.
Method of approach	11	How were participants approached? e.g. face-to-face, telephone, mail, email	p.7: All participants took part in a telephone survey.
Sample size	12	How many participants were in the study?	p.7: N=93 participants
Non-participation	13	How many people refused to participate or dropped out? Reasons?	p.10: The follow-up calls examined here were implemented in the course of the study. From the time follow-up calls were introduced in the main study, all participants took part in the survey.

Setting

Setting of data collection	14	Where was the data collected? e.g. home, clinic, workplace	p.7: The data was collected at home or at the university.
Presence of non-participants	15	Was anyone else present besides the participants and researchers?	p.7: The interviewers were always alone in the rooms concerned.
Description of sample	16	What are the important characteristics of the sample? e.g. demographic data, date	p.9: The sample is described in table 1.

Data collection

Interview guide	17	Were questions, prompts, guides provided by the authors? Was it pilot tested?	Appendix 1 contains the template for the follow-up calls.<br>Appendix 3 contains the coding guide.<br>P.7: The study wasn´t pilot tested.
Repeat interviews	18	Were repeat interviews carried out? If yes, how many?	No, no interviews were repeated.
Audio/visual recording	19	Did the research use audio or visual recording to collect the data?	p.7: The telephone calls were not recorded. However, a memory log was created in each case.
Field notes	20	Were field notes made during and/or after the interview or focus group?	p.8: Memos were created continuously during the coding rounds, and the entire analysis process was documented in a research logbook.
Duration	21	What was the duration of the interviews or focus group?	p.9: The interviews lasted between 25 and 40 minutes.
Data saturation	22	Was data saturation discussed?	p.7: Since all available protocols were included in the analysis, saturation can be assumed.
Transcripts returned	23	Were transcripts returned to participants for comment and/or correction?	p.7: No, the transcripts were not returned to participants for comment and/or correction.

Domain 3: analysis and findings

Data analysis

Number of data coders	24	How many data coders coded the data?	p.8: AB coded all protocols; CF performed the counter-coding of seven protocols.
Description of the coding tree	25	Did authors provide a description of the coding tree?	Yes, appendix 3 contains the coding tree.
Derivation of themes	26	Were themes identified in advance or derived from the data?	p.8: Deductive and inductive approaches were combined to develop the category system.
Software	27	What software, if applicable, was used to manage the data?	p.8: We used MAXQDA (VERBI-Software).
Participant checking	28	Did participants provide feedback on the findings?	No, participants didn´t provide feedback on the findings.

Reporting

Quotations presented	29	Were participant quotations presented to illustrate the themes/findings? Was each quotation identified? e.g. participant number	p. 10-17: All findings were supported by identifiable citations.

Data and findings consistent	30	Was there consistency between the data presented and the findings?	Yes, see tables 2-4.
Clarity of major themes	31	Were major themes clearly presented in the findings?	Yes, see section 3.1, 3.2 and 3.3.
Clarity of minor themes	32	Is there a description of diverse cases or discussion of minor themes?	Yes, even topics that were rarely mentioned are included in the presentation.
