# Supplementary material for: Feasibility of a conversation-based brief intervention in general practice to reduce post-traumatic symptoms after intensive care treatment—A qualitative analysis of the PICTURE study
Source: PLOS Ment Health. 2026 Jan 28;3(1):e0000467. doi: 10.1371/journal.pmen.0000467 (PMC12851445; doi:10.1371/journal.pmen.0000467)
Supplement: S4 Data — (PDF) [file pmen.0000467.s004.pdf]

List of codes

List of codes	Frequency
Code system	1554
Feasibility	0
Session 1	0
1_duration	50
1_Theme	42
1_Problems	45
1_well done	38
1_Acceptance by patient	73
Session 2	0
2_Duration	46
2_Theme	53
Traumatic memories of the ICU	67
2_Problems	87
2_well done	37
2_Acceptance by patient	66
Statement on the tasks of medical assistants	12
Acquisition of methodological skills	0
Preparation through training	59
Download link and DVD used	38

Repetition of the training content	44
Problems, fears, doubts	20
Self-doubt regarding the implementation	13
Suggestions for improvement	22
Effectiveness	0
adverse effects	5
beneficial	29
obstructive	16
Observations from GPs	18
Strengthening the therapeutic relationship	9
Statements from patients	57
Being allowed to talk	35
Physical condition	24
PTSD symptoms continue	49
Meaningfulness / transferability	0
Meaningfulness given	37
Help in everyday life	13
Well feasible	8
Demand given	7
Meaningfulness not given	5
Implementability in everyday practice	0
unreservedly "Yes"	27

Fears not confirmed	8
with restrictions: "yes, but"	32
not conceivable, "no way"	11
Suggestions for prospective implementation	11
limiting factors	10
Remuneration	18
Time required	55
Aspects of the pandemic	6
Comments on participation in the study	26
Gaining knowledge, raising awareness of the topic	45
Acceptance of general practitioners, self-awareness	14
Nomination of further patients for study participation	11
Further comments	0
Topic of the 3rd session	57
Patient was not known in advance	15
Doubts about inclusion diagnostics	28
NET is not the right therapeutic approach	6
General practitioner has previous psychotherapeutic knowledge	10
Patinet has previous therapy experience	20
high performer	15
low performer	5
