# Supplementary material for: Feasibility of a conversation-based brief intervention in general practice to reduce post-traumatic symptoms after intensive care treatment—A qualitative analysis of the PICTURE study
Source: PLOS Ment Health. 2026 Jan 28;3(1):e0000467. doi: 10.1371/journal.pmen.0000467 (PMC12851445; doi:10.1371/journal.pmen.0000467)
Supplement: Attachment — Submitted filename: Response-to-reviewers.docx [file pmen.0000467.s006.docx]

**Point-to-point reply**

Ref. No.: PLOS Mental Health PMEN-D-25-00399

Title: " Feasibility of a conversation-based brief intervention in general practice to reduce post-traumatic symptoms after intensive care treatment - a qualitative analysis of the PICTURE study”.

**Journal Requirements:**

1. Please send a completed 'Competing Interests' statement, including any COIs declared by your co-authors. If you have no competing interests to declare, please state "The authors have declared that no competing interests exist".

- Response: The authors have declared that no competing interests exist.

2. Please amend your detailed Financial Disclosure statement. This is published with the article. It must therefore be completed in full sentences and contain the exact wording you wish to be published.

If you did not receive any funding for this study, please simply state: “The authors received no specific funding for this work.”

- Response: Funding: This study was funded supported by the German Research Foundation (DFG Grant: GE 2073/8-1). Our thanks also go to the German Center for Mental Health (DZPG), the Bavarian Research Practice Network (BayFoNet) and the Research Practice Network Berlin-BrandenburgThuringia (RESPoNsE). The funder had no role in the design and conduct of the study; collection, management, analysis and inter-pretation of the data; preparation and review or approval of the manuscript, or the decision to submit the manuscript for publication.

3. We note that you have indicated that there are restrictions to data sharing for this study. For studies involving human research participant data or other sensitive data, we encourage authors to share de-identified or anonymized data. However, when data cannot be publicly shared for ethical reasons, we allow authors to make their data sets available upon request. For information on unacceptable data access restrictions, please see http://journals.plos.org/plosone/s/data-availability#loc-unacceptable-data-access-restrictions.

Before we proceed with your manuscript, please address the following prompts:

a) If there are ethical or legal restrictions on sharing a de-identified data set, please explain them in detail (e.g., data contain potentially identifying or sensitive patient information, data are owned by a third-party organization, etc.) and who has imposed them (e.g., a Research Ethics Committee or Institutional Review Board, etc.). Please also provide contact information for a data access committee, ethics committee, or other institutional body to which data requests may be sent.

- Response: Data will be made available on request. For enquiries about data use, potential collaborations or related projects, interested researchers are encouraged to contact the principal investigator of the study Jochen Gensichen.

**Comments from reviewers:**

**Reviewer 1:**

General comments:

Introduction

The first part of the introduction is actually good, allowing the statement of the importance of the intervention under study, nevertheless, the flow in the second part is lost as there is a lack of:

Definition of what is trauma-focused narrative exposure therapy is and a brief literature review on its uses and effectiveness. I could not follow the argument until I looked in the literature for the NET and Picture study.

The following information would have been interesting and useful for the reader that is not familiar with this work, like the paragraph in the following article: Sanftenberg, L., Beutel, A., Friemel, C.M. et al. Barriers and opportunities for implementation of a brief psychological intervention for post-ICU mental distress in the primary care setting – results from a qualitative sub-study of the PICTURE trial. BMC Prim. Care 24, 113 (2023). https://doi.org/10.1186/s12875-023-02046-0

(Therefore, the PICTURE trial (“PTSD after ICU survival”) has been initiated to improve the follow-up care of ICU patients in primary care by testing a brief talking therapy with case management. The intervention is based on narrative exposure therapy (NET), a well-established combination of testimony therapy and cognitive-behavioral therapy, originally developed for low-income countries [17]. Assuming a disturbed memory formation for a traumatic event the NET aims to rebuild memory by a detailed and chronological review of the traumatic scenario in which fragmented trauma memories can be embedded. By using targeted questioning the therapist facilitates a vivid narration and mental re-exposure to the traumatic event in the patient.

- Response: We thank the reviewer and fully agree that there was insufficient information provided here and have supplemented it (see section 1.2).

Please include a review about the existing experience in the application of this tool and benefits according to the literature.

- Response: We have followed this important advice and included the relevant literature (see section 1.2).

Additionally, the literature should describe potential factors that may influence the implementation and effectiveness of this program, allowing for later evaluation of the results in the discussion.

- Response: As this intervention is novel, no citable literature is yet available. This study presents initial findings on its implementation.

Methodology: well described, comprehensive, and includes the elements needed for qualitative study methodology reporting.

However, the study does not say where the questions came from or how the interview questions were created. How was it decided?

- Response: Thanks for pointing that out. We have added this information in the text (please see also details below).

Correct the word” seven tra7nscripts” line 150.

- Response: We thank the reviewer for the careful examination and corrected the word.

Results

Categories that have 0 frequencies do not need to be reported.

- Response: Categories with a frequency of 0 are not reported.

For reflection: what is the point of integrating sociodemographic variables when you do not incorporate them in the explanation of the results? For example, were there differences in the acceptance of the opinion about feasibility according to years of experience, for example???

- Response: Thank you for this important feedback. Following the comment of the reviewer, we revised and condensed the table while retaining the sociodemographic information, as we consider it essential for readers to understand the population from which the sample was drawn

Discussion: what about comparing your results with similar studies made under similar constructs and conditions, more in-depth, in a more reflective way, not only saying it is similar or not to other studies, but also how it is similar and different and what it means for the German context?

- Response: Our studies present a novel treatment approach for which no prior empirical investigations exist.

It would be interesting to have a chart that explains the main codes generated and themes based on codes in order to follow how those who have analyzed the data have come up with the codes.

- Response: Following the reviewer’s comment we have clarified the explanation of how the codes are generated in the text and refer to the files attached in the appendix in this context (Appendix-1_Template_follow-up-calls and Appendix-3_Codebook).

**Reviewer 2:**

General comments:

Best authors. Thank you very much for submitting your manuscript to PLOS Mental Helath and that I was allowed to review it. Its theme is highly interesting, as many ICU survivors do suffer from longterm consequences that form PICS, comprising mental helath symptoms of PTSS and in some, also developing into PTSD. It is true that there are numerous ICU survivors who because of their post-ICU state, needs more help, and GPs are one of many groups of professionals who meet and treat these patients. GPs expressions are absolutely interesting to know more about. There is a need for more to be done and I salute your interest in the field and work done yet. Keep going on.

- Many thanks for your encouraging words!

However, I consider that there are some major issues with the manuscript that needs to be handled before it can be re-considered. To my mind, it needs additional rounds of work by the whole group of authors, so that the rationale behind this qualitative sub-study are easier to follow, how the results relates to eachother, the meaning of the results in each cathegory and why you choose to present them and the the citations used to examplify each cathegory, also to better discuss and present the clinical impact of your findings. As post-ICU procedures may be very different in Germany than other countries such as Australia, England or others, a suggestion is if your manuscipt would benefit to be submitted to a German native paper? I wish you all good.

- Response: We appreciate the reviewer’s important comments. We deliberately chose ***PLOS Mental Health*** because it is an international journal with a global readership, allowing findings to be considered in a broader context. Publishing in an international outlet also facilitates comparisons across countries, which we believe is particularly valuable for this topic.

1) There are no red thread throughout the manuscript and the logic behind introduction, method, results, discussion, and conclusion are not clear. The rationale behind this study is not clarly stated, and methodological issues are not developed (only slightly mentioned) and they are not discussed.

- Response: We thank the reviewer for this important feedback. We understand the concern regarding the clarity of the manuscript’s overall structure and rationale. In response, we have revised several sections to strengthen the logical flow and ensure a clearer “red thread” throughout the paper. Specifically, we have (1) expanded the introduction to more explicitly articulate the rationale and objectives of the study, (2) elaborated the methodological procedures to provide greater transparency and detail, (3) improved the organization of the results to align more clearly with the research questions, and (4) revised the discussion to more explicitly relate the findings back to the study aims and methodological considerations. We believe these changes significantly enhance coherence and readability.

2) Spelling is not correct, and it seems to me that it has not been proofread. There is inconsistency throughout the manuscript, meaning, because of poor spelling and lack of structure in the text, it is very difficult to understand what the authors try to say.

- Response: Many thanks for considering checking the spelling of the manuscript. We have carefully examined the spelling and hopefully completed all corrections.

3) It seems to me that this text is partly AI written, and if so, the use of AI is not stated by the authors. To me as a researcher, this would be unethical.

- Response: See, if you find spelling errors and find that parts are difficult to understand, it is already clear that the manuscript has been written by a non-native speaker and that the text was not generated by AI.

4) The logic behind the results section is not clear. Tables are difficult to understand and the do not reflect the expressions in the results section.

- Response: Thank you for pointing this out. We hope that the edited tables are now easier to read and understand.

5) In the results section, citations seems to just be written out and is is not clear why all are spelled out in each section, it seems there are no clear rationale why the content in each cathegory has been put just there. There are sections where citations are just spelled out, one after another, but without any logic how they relate to each other. Also, many of the sitations seems to be from patiens and not by GPs (as this manuscript states to aim and to reflect!). This major issue, also makes me to think that this manuscript is AI written? or in best case, not worked with.

- Response: We are somewhat surprised by this statement. Our intention was to support the presentation of the results per paragraph with appropriate quotations and then summarize them briefly in a table. We also cannot understand the assumption that the quotes come from patients and not from GPs, as this is mentioned several times in the text.

6) The manuscript do not fully follow authors guideline.

- Response: We hope that the revised manuscript meets all requirements.

7) References used, are old and not up to date, they are few, results are not discussed against up-to-date relevant literature. References are not cited correctly according to guideline.

- Response: We wonder which references the reviewer is referring to. We have included recent references. And we corrected the citation as requested.

8) COREQ checklist can be better filled in, as the topics are not clearly stated in the text.

- Response: Thanks for pointing that out. We have updated the COREQ checklist.

9) The discussion seems not to be about the results. And no up to date and relevant references are used.

- Response: See point 1 and 7.

10) The conclusion is hard to follow and to my mind, do not reflect the study objective, results and discussion.

- Response: We have reread the manuscript and clarified points that seem difficult to read.

11) There are no declarations of who of the authors have done what in this manuscript.

- Response: We thank the reviewer for this important point. We have included this information under “authors´ contributions.”
